# Case report: Autoimmune hemolytic anemia caused by warm and cold autoantibodies with complement activation—etiological and therapeutic issues

**DOI:** 10.3389/fped.2023.1217536

**Published:** 2023-09-19

**Authors:** Daniel Turudic, Sara Dejanovic Bekic, Lucija Mucavac, Maja Pavlovic, Danko Milosevic, Ernest Bilic

**Affiliations:** ^1^ Department of Pediatric Hematology and Oncology, University Hospital Centre Zagreb, Zagreb, Croatia; ^2^ Croatian Academy of Medical Sciences, Zagreb, Croatia; ^3^ Department of Pediatrics, Zabok General Hospital and the Croatian Veterans Hospital, Zabok, Bračak, Croatia; ^4^ School of Medicine, University of Zagreb, Zagreb, Croatia

**Keywords:** AIHA, warm antibodies, cold antibodies, complement, child

## Abstract

**Introduction:**

Research on mixed warm and cold autoantibodies in autoimmune hemolytic anemia (AIHA) targeting erythrocytes [red blood cells (RBCs)] and platelets is scarcely reported.

**Case presentation:**

In this study, we present the case of a 5-year-old boy with positive direct [anti-IgG (1+), anti-IgG-C3d (3+)], and indirect antiglobulin (Coombs) tests. The RBCs were coated with polyspecific-positive, warm IgG autoantibodies alongside activated complement components. Plasma-containing immunoglobulin M (IgM) class autoantibodies were found in 1:64 titers with a wide temperature range of 4°C–37°C. The platelets were also coated with IgM autoantibodies. There was a reduction in the levels of the classical and alternative complement pathways, such as C3, C4, ADAMTS13 metalloprotease activity, factor H antigen, complement factor B antigen, and C1q antigen alongside the elevated sC5b-9 terminal complement complex. Hematuria and/or proteinuria, reduced diuresis, and elevated levels of serum creatinine were absent. The kidney ultrasound report was normal. A recent combination of Epstein–Barr virus (EBV) and cytomegalovirus (CMV) infection was found. The first-line treatment consisted of intravenous methylprednisolone [4 mg/kg/body weight for the first 72 h (q12 h), followed by 2 mg/kg body weight for 21 consecutive days with a slow steroid reduction until plasmapheresis (PLEX)]. After the patient showed limited response to corticosteroid therapy, rituximab (375 mg/m^2^) was administered once a week (five doses in total), with vitamins B9 and B12. These strategies also showed limited (partial) therapeutic benefits. Therefore, the treatment was switched to PLEX (five cycles in total) and intravenous immunoglobulin (IVIg) (1 g/kg/5 days). This combination significantly improved RBC count and platelet levels, and C3 and C4 levels returned to normal. A follow-up of 2.5 years after treatment showed no sign of relapse. A genetic analysis revealed a rare heterozygous intronic variation (c.600-14C > T) and heterozygous Y402H polymorphism of the CFH gene. c.600-14C > T mutation was located near the 5′ end of exon 6 in the gene encoding the complement C3 protein of unknown significance. We presumed that the complement regulators in our patient were sufficient to control complement activation and that complement blockade should be reserved only for devastating, life-threatening complement-related multiorgan failure.

**Conclusion:**

We believe that EBV and CMV triggered AIHA, thus activating the complement cascade. Hence, we used corticosteroids, rituximab, vitamins B9 + B12, PLEX, and fresh frozen plasma (FFP) as treatment. Final remission was achieved with PLEX and FFP. However, an additional late effect of B12 rituximab and the disappearance of long-lived circulating plasma cells should not be completely ignored. Complement activation with a genetic background should be assessed in severe warm and cold hemolytic anemias caused by autoantibodies.

## Introduction

Research on the simultaneous combination of warm and cold autoantibodies in autoimmune hemolytic anemia (AIHA) targeting erythrocytes [red blood cells (RBCs)] and platelets was first published almost five decades ago (1, 2). There are no firmguidelines for the treatment for this combination of hematological autoimmune diseases. In this study, we present a case wherein we treated a child with the aforementioned combination of diseases that required a personalized treatment approach.

## Case presentation

A 5-year-old boy with a life-threatening severe macrocytic hemolytic anemia was admitted in our hospital. He had signs of ongoing hemolysis with elevated total bilirubin, lactate dehydrogenase (LDH), and complement activation upon admission. Initial counts of hemoglobin, hematocrit, and platelet were reported alongside low counts of haptoglobin and elevated mean corpuscular volume (MCV) and reticulocyte. Classical and alternative complement pathways such as C3, C4, ADAMTS13 metalloprotease activity, factor H antigen, complement factor B antigen, and C1q antigen showed reduced levels. ADAMTS13 activity was decreased but not deficient. The elevated level of sC5b-9 (terminal complement complex) revealed the formation of a complement-mediated membrane attack complex (MAC) (Table 1). Hematuria and/or proteinuria, reduced diuresis, or elevated serum creatinine were absent. The ranges of urea and creatinine were within reference values. The kidney ultrasound was normal. An immunohematological analysis upon admission showed AIHA with positive direct [anti-IgG (1+), anti-IgG-C3d (3+)] and indirect antiglobulin (Coombs) tests. The RBCs were coated with polyspecific-positive, warm IgG autoantibodies alongside activated complement components. Plasma-containing IgM class autoantibodies were found in 1:64 titers with a wide temperature range of 4°C–37°C. The rhesus phenotype was CcDEe with K (−), Jka (−), and Jkb (+). Platelets were also coated with IgM autoantibodies. A probable trigger was a recent combination of Epstein–Barr virus (EBV) and cytomegalovirus (CMV) infection (Table 2).

**Table 1:** Relevant initial laboratory, microbiological, and complement pathway testing results.

Complete blood count	Onset	Relapse	Before PLEX	After PLEX	Reference interval
Red blood cells (RBCs)	**1.32**	**1.57**	**2.25**	4.10	4.00–5.00 × 10^12^
Hemoglobin	**44**	**53**	**76**	134	109–138 g/L
Hematocrit	**14.9**	**16**	**25.2**	38.3	32%–40.4%
Mean corpuscular volume (MCV)	**112.6**	**101.8**	**112.1**	**93.4**	73.8–89.4 fL
Mean corpuscular hemoglobin (MCH)	**33.3**	**33.8**	**33.8**	**32.7**	24.3–29.2 pg
Mean corpuscular hemoglobin concentration (MCHC)	296	332	301	350	300–350 g/L
Red blood cell distribution width (RDW)	**37.7**	**34.1**		15.4	11.9–16.2%
Reticulocytes *per mile*	**158.7**	**162.6**	**261.5**	**19.9**	4.0–19.0/1,000 RBCs
Reticulocytes (absolute count)	**410**	**260**	**590**	82	20–94
Platelets	**110**	302	151	310	150–450 × 10^9^/L
Biochemistry					
Erythrocyte sedimentation rate	4				0–20 mm/h
Total bilirubin	**24**		**24**	7	<20 µmol/L
Uric acid	3.3	3.2	2.5	1.9	1.8–6.0 mmol/L
Creatinine	19	20	19	23	25–42 µmol/L
Aspartate aminotransferase (AST)	30	25		25	24–49 U/L
Alanine aminotransferase (ALT)	19	**27**	** **	**22**	9–20 U/L
Gamma-glutamyl transferase (GGT)	60	**56**	** **	**71**	4–22 U/L
Lactate dehydrogenase (LDH)	**411**	**372**		201	150–300 U/L
Vitamin B12	203		**130**		145–637 pmol/L
Folic acid	**6.8**		**>45.0**		8.8–39.7 nmol/L
G6PD	**627**		** **		120–299 mU/Er
Haptoglobin	**0.01**		**<0.10**		0.3–2.0 g/L
Complement pathway					
ADAMTS13 metalloprotease activity	**38%**				67%–150%
Total complement activity and classical pathway	**0**				48–103 CH50/mL
Total complement activity and alternative pathway	**1%**				70%–105%
Complement C3	**0.64**				0.9–1.8 g/L
Complement C4	**0.02**				0.15–0.55 g/L
Factor H antigen	**84**				250–880 mg/L
Complement factor I antigen	81%				70%–130%
Complement factor B antigen	**64%**				70%–130%
Antifactor H IgG autoantibody	78				<110 AU/mL
C1q antigen	**28**				60–180 mg/L
Anti-C1q IgG autoantibody	1				<52 U/mL
sC5b-9 (terminal complement complex)	**301**				110–252 ng/mL
Immunology					
ANA (antinuclear antibody)	Negative				Negative
ENA (extractable nuclear antigen antibodies)	Negative				Negative
Lupus anticoagulant	Negative				Negative
Antineutrophil cytoplasmic antibodies (ANCAs)	Negative				<1:20
Miscellaneous					
Methylmalonic acid (serum, plasma)	**0.66**				<0.51
Homocysteine	13.1				<15 µmol/L

The bold values indicate numerical values outside of standard reference range.

**Table 2:** Microbiology panel.

Name	Result	Interpretation	Reference value
Parvovirus B19 IgM	1.80	Negative	<8.5 U negative
Parvovirus B19 IgG	3.90	Negative	<8.5 U negative
Mycoplasma pneumoniae IgM	2.10	Negative	<8.5 U negative
Mycoplasma pneumoniae IgG	3.10	Negative	<8.5 U negative
CMV IgM	0.03	Negative	<0.70 negative
CMV IgG	32.00	**Positive**	≥6 aU/mL positive
EBV VCA IgM	0.00	Negative	≤0.11 negative
EBV VCA/EA IgG	4.40	**Positive**	≥0.21 positive
EBV EBNA IgG	7.88	**Positive**	≥0.21 positive
Rubella IgM	0.08	Negative	<0.80 negative
Rubella IgG	1.00	Negative	<10 IU/mL negative
Anti-HCV	0.07	Negative	<1.00 negative

The bold indicates outside of normal reference ranges.

Malignant hematological disorders (bone marrow biopsy), folate deficiency anemia, vitamin B12 deficiency, homocystinuria, and paroxysmal nocturnal hemoglobinuria (PNH) were excluded (Table 1). Anti-nuclear antibodies, extractable nuclear antigen antibodies, rheumatoid factor, and lupus anti-coagulant screens were negative. Quantitative flow cytometry showed a negative result for PNH cells. A bone marrow biopsy showed dyserythropoiesis, with predominantly normoblastic RBCs and a few megaloblastic RBC forms with nuclear budding and karyorrhexis. An initial immunophenotyping test revealed a reduced number of T cells (4%) and B cells (8%) with a very low mitotic index found by cytogenetic karyotyping. The absolute count of CD3 (1,155 cells/µL), CD3 + CD4+ (440 cells/µL), and CD3-CD16 + CD56+ (natural-killer cells, 140 cells/µL) was low with a reduced CD4/CD8 ratio (0.8).

Prior to treatment, a bone marrow biopsy was performed to exclude malignant hematological disorders. As we were aware of the simultaneous presence of warm and cold AIHA autoantibodies, immunosuppressive therapy was started alongside periodic packed RBC and platelet substitutes, avoiding such substitution as much as possible (3, 4). The first-line treatment consisted of intravenous methylprednisolone [4 mg/kg/body weight for the first 72 h (q12 h), followed by 2 mg/kg body weight for 21 consecutive days and very slow steroid reduction until plasmapheresis (PLEX)]. PLEX was performed by using a central double lumen catheter. Approximately 1–1.5 plasma volume was swapped with normal saline and fresh frozen plasma. Periodic packed RBC and platelet transfusions were administered as needed (4, 5). We maintained the hemoglobin count at 70–80 g/L and the platelet count at 30–50 × 109 to avoid giving too much “fuel” to the hemolysis (4, 6).

After the corticosteroid therapy, a follow-up immunohematological analysis still showed positive results for both indirect and direct Coombs tests [anti-IgG (3+), anti-IgA (1+), anti-IgM (3+), and anti-C3c (3 + s)]. RBCs were coated with a large amount of polyspecific IgG class autoantibodies (subclasses IgG1 and IgG3, IgM, and IgA) and activated complement components (decreased C3 and C4). Cold antibodies of the IgM class were still present in the plasma in titer 1:64 with a normal temperature range of 4°C–20°C alongside warm antibodies of the IgG class.

**Figure 1:**
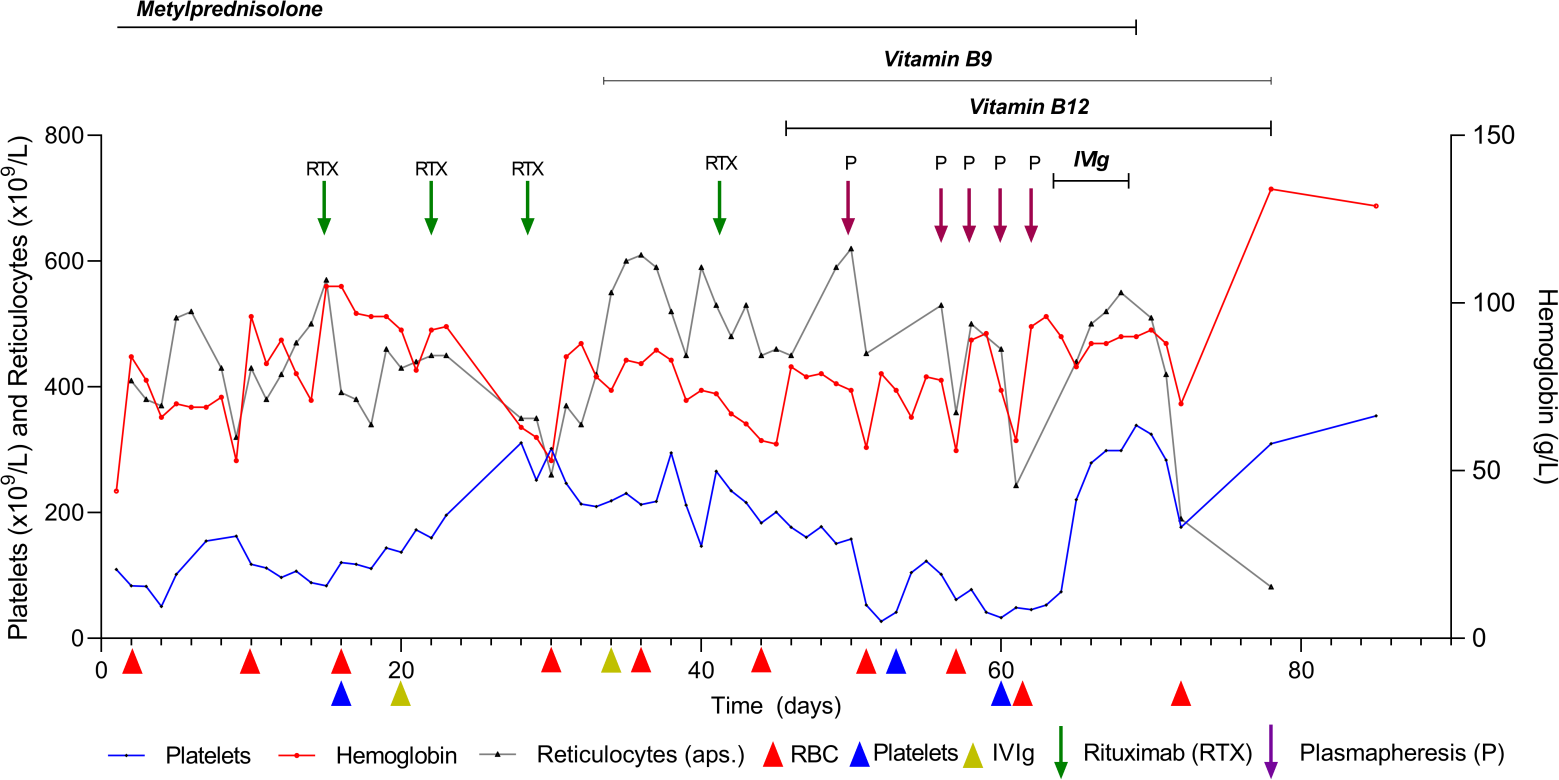
Patient follow-up flowchart.

After the limited response to corticosteroid therapy, probably due to the reduction but not the removal of warm autoantibodies, rituximab (375 mg/m^2^) was administered once a week (five doses in total), vitamin B9 (1 mg) was administered per day, and vitamin B12 (1,000 mcg) was intramuscularly administered once a day for 7 days and then once a week for 1 month. These strategies also exhibited a limited (partial) therapeutic benefit, as the child was further dependent on periodic pack RBC and platelet transfusions. Therefore, the treatment was switched to PLEX (five cycles in total, with side effects such as an increased tendency to clotting) and IVIg (1 g/kg/5 days) with vitamins B9 and B12. This combination of medicaments significantly improved RBC count and platelet levels (Figure 1), and C3 and C4 levels returned to normal. The follow-up of 2.5 years after the treatment reported no sign of relapse.

During follow-up, the patient received levetiracetam (40 mg/kg per day divided into two doses) and phenobarbitone (5 mg/kg divided into two doses) treatment for MRI- and EEG-verified epileptic encephalopathy. Prolongued phenobarbitone therapy can also interfere with the absorbtion of vitamins B9 and B12, which indicates their supplementation necessary. Furthermore, a gene panel analysis found a heterozygous frameshift variant of the *CNTNAP2* gene associated with developmental delay and epilepsy. The patient’s family history was negative for any immune or complement-mediated diseases, and therefore, parental genetic testing was not performed.

In addition, the genetic analysis revealed a heterozygous mutation for a rare intronic variation (c.600-14C > T) and heterozygous Y402H polymorphism of the CFH gene. The c.600-14C > T mutation was located near the 5′ end of exon 6 in the gene encoding the complement C3 protein (C3). This rare variation was previously described in one patient with atypical hemolytic uremic syndrome (aHUS), in one patient with glomerulonephritis, in one subject suffering from C3 deficiency, and in one patient with age-related macular degeneration. This rare variation was also identified in healthy subjects with a relatively low frequency (0.04%–1.1%), but no functional studies have been conducted on its possible role. However, the patient is a carrier of a “variant of unknown significance” in the C3 gene, but further analysis (control of the patient and family members) would be necessary to make an even stronger conclusion of whether this intronic C3 has any influence on gene expression.

## Discussion

We presume that predisposing genetic factors with strong triggers (EBV, CMV) generated warm (IgG-mediated) and cold (IgM-mediated) autoantibodies lead to direct cell lysis and antibody-dependent cellular cytotoxicity (ADCC). Both IgM (cold) autoantibodies and IgG (warm) autoantibodies can bind with C1, triggering the classical complement pathway, which stimulates combined intravascular and extravascular hemolysis (7). It is a known fact that when RBCs encounter such inflammatory attacks, they may undergo apoptosis-like programmed cell death (eryptosis) (3, 8–10).

A review of the literature revealed limited results on various etiological and therapeutic approaches. EBV infections were significantly associated with cold IgM-mediated AIHA (rarely with warm IgG agglutination) (11), while CMV infection is associated mainly with warm IgG-mediated AIHA. Given that EBV and CMV infections are the most probable causes of disease triggers, three possibilities arise:

(1) EBV and CMV trigger AIHA, and the complement cascade acts simultaneously but independently. No connection exists between AIHA and complement activation. Since we did not find EBV/CMV IgM but only IgG, such late infection–triggering complement activation in already overcome infections at the full-blown AIHA seems improbable.
(2) EBV and CMV trigger complement activation, which further triggers AIHA (complement-mediated). In such cases, we may expect the eventual onset of thrombotic microangiopathy [thrombotic thrombocytopenic purpura (TTP), HUS, and aHUS] in some patients with warm or cold autoantibodies, which individually are not that rare. The absence of such clinical features and the limited effect of eculizumab (extravascular hemolysis remains unaffected by the treatment with eculizumab), with a modest anemia reduction after its administration, speak against this event cascade. In the DECADE trial, the cold agglutinin titer alongside the intensity of coated C3d RBCs in a wide temperature range remained (12).
(3) EBV and CMV trigger AIHA, which, in turn, activates the complement cascade. Infection-triggered complement-driven exacerbation of hemolysis is common for cold autoantibodies (13). Such activation will eventually cease with sufficient complement regulators in the affected individual. Therefore, complement activation will ultimately break off with successful AIHA treatment without permanent organ damage. In our patient, the kidneys were affected but remained asymptomatic.

We discuss the possible cascade of the aforementioned possibility #3. In AIHA, increased destruction of RBC by anti-RBC autoantibodies either via direct cell lysis, complement activation, or ADCC formation leads to the release of toxic hemolysis-derived products such as cell-free hemoglobin and heme.

• Overabundance of cell-free hemoglobin increases free iron and pro-oxidative reactive oxygen species (ROS). These highly reactive molecules oxidize proteins, lipids, and DNA—leading to cell damage and ferroptosis (14). The time- and concentration-dependent increase in endothelial aggressors as a consequence of antibody-mediated RBC hemolysis (hemoglobin and heme) acted as a second-“hit” phenomenon, causing tissue and organ injury by triggering prothrombotic and proinflammatory pathways (sC5b-9). We concur with the *rationale* of the second-hit hypothesis for such mixed warm and cold antibody AIHA.
• Extracellular heme has pro-oxidative, proinflammatory, and prothrombotic properties via interaction with the complement system, immunoglobulins, and circulating hemostatic plasma proteins. The interaction of heme with the complement components can activate the alternate complement pathway either via C1q inhibition or via C3 activation, forming an overactive C3/C5 convertase (15, 16). The brief exposure of endothelial cells to heme decreases the expression of complement regulators, such as membrane cofactor protein MCP (CD46) and decay-accelerating factor CD55, providing factor H a key role in complement regulation. In addition, free heme promotes rapid exocytosis of Weibel–Palade bodies with a membrane expression of P-selection (17). Physiologically, heme is neutralized in intravascular space by haptoglobin and hemopexin, and HO-1 (heme oxygenase-1) in intracellular space. Based on what we encountered in our patient with massive hemolysis, these defensive mechanisms were overpowered by the ongoing overwhelming hemolysis (18).
• P-selection serves as an anchoring platform for the C3b and C3 activating alternate pathways and releasing the ultralarge von Willebrand factor (UL-vWF). Heme also inhibits the cleavage of the UL-vWF by ADAMTS13. However, decreased ADAMTS13 did not correspond to TTP, as the ADAMTS13 level was too high (ADAMTS13 ≤ 10). However, such a level of ADAMTS13 corresponds with other thrombotic microangiopathies (typical/atypical hemolytic uremic syndromes). The resulting excess of UL-vWF induces platelet activation and aggregation by spontaneously binding to platelets or interacting with platelet glycoprotein Ib (GPIb) (16).

The formation of C3b is a focal point in the activation of three complement pathways, namely, classical, alternate, and lectin. IgM-mediated cold agglutinins on the RBC surface activate C1q, thus initiating the classical pathway. C3 convertase is attached to surface C3b, which, in turn, activates the C5 convertase and lectin complement pathway. In the event of massive complement overactivation and consumption, the alternative pathway can activate C3 production, generating alternative C3 (C3bBb) alongside classical and lectin C3 (C4b2a) convertases (19). These convertases generate additional C3b and C5 convertases that are responsible for complement cascade, opsonization, and inflammation. By binding opsonin C3b on the RBC surface, factor H regulates the inhibition of C3 and C5 convertase production. In theory, the reduction of factor H level increases the convertase production into sC5b-9 (MAC) (20). In cold agglutinin disease, the levels of complement C3 and, in particular, C4 are often reduced, reflecting continuous consumption (20). Reduction of C3 and C4 levels may also be caused by infection-related consumption (4). In addition, heterozygous gain-of-function C3 mutations can increase the formation of C3bBb (21).

We performed aggressive prolonged corticosteroid therapy on a life-threatening AIHA (3, 22, 23) (Figure 1). Mixed cold and warm AIHAs generally require two or more therapy lines with corticosteroids, such as rituximab, immunosuppressants, and/or splenectomy (23–25). We also considered the possibility of the patient having atypical AIHA due to significantly positive warm IgM (3+) and weakly positive IgA (+) antibodies. A positive direct antigen test and still inconclusively elevated IgA speak against this possibility (23). After the unsatisfactory effect of the corticosteroid therapy, we introduced rituximab (not combined with bendamustine) because the patient was too frail to sustain both medicaments, alongside vitamin B12 and B9 substitutions. Vitamin B9 + B12 substitution was also beneficial for megaloblastic dyserythropoiesis. A long-term follow-up of the patient without disease relapse proved the effectiveness of IVIg therapy with B12 + B9. The follow-up consisted of a complete blood count with reticulocytes and platelets, indirect bilirubin, AST, LDH, haptoglobin, and occasionally C3, C4, and sC5b-9 levels alongside indirect and indirect Coombs tests. We overruled the presence of spurious macrocytosis and agglutination in the blood samples, which may occur when the sample is cooled during processing, as well as the RBC count, which may be artifactually low due to the presence of macroaggregates with an inappropriately high MCV (26, 27).

Due to the ongoing hemolysis, a different therapeutic approach was devised. Accordingly, we initiated PLEX and IVIg therapy, hoping that it would be sufficient to control the disease (8, 23, 28, 29). It proved to be a successful decision to take. The rapid normalization of LDH and bilirubin is probably partly explained by plasma exchange, replenishing ADAMTS13 and removing ULVWF and ADAMTS13 inhibitors/proteolytic inactivators, such as ADAMTS13 autoantibodies, IL-6, plasma-free hemoglobin, plasmin, thrombin, and granulocyte elastase (30). The absence of reduced diuresis, hematuria, uremia, and diarrhea makes the typical (HUS) or atypical (aHUS) hemolytic uremic syndrome diagnosis unlikely. Nevertheless, we decided to ascertain the complete genetic and complement background.

The aforementioned clinical and laboratory presentation makes us consider these complement activations as hemolytic–uremic-like syndromes. Therefore, a complement blockade was considered an option. As we had already partially controlled AIHA, we decided not to introduce a complement blockade. Complement cascade inhibitors in AIHA (mediated by warm or cold antibodies) such as sutimlimab (anti-C1), Berinert (C1 inhibitor), or pegcetacoplan (anti-C3) have been used in selected patients unresponsive to B-cell therapies or severe hemolytic anemia requiring rapid response to severe hemolysis (4, 31–33). In AIHA with warm and cold autoantibodies, C1q binds autoantibodies on RBCs, causing the classical pathway activation by serine proteases C1r and C1s, thus leading to C3b deposition (4). Complement blockade therapy (CBT) with C1 inhibitor Berinert revealed an immediate effect (increase in hemoglobin levels) by C3d deposit reduction. However, it failed to decrease hemolytic activity, stabilize transfusion efficacy, or reduce systemic complement activation (4, 31). Sutimlimab (anti-C1-humanized monoclonal antibody) inhibits the classical complement system by binding to complement protein subcomponent 1 (sC1). By inhibiting C1, only the classical complement pathway is affected, whereas lectin and alternative pathways remain intact. In theory, patients with only C1 inhibition will still generate C3a and C5a anaphylatoxins, which is an advantage offered by sutimlimab as against pegcetacoplan (c3). Sutimlimab has exhibited a favorable response to complement-mediated AIHA by preventing the deposition of opsonins on the surface of RBCs (20, 32–33). This promising result of sutimlimab in warm AIHA requires further verification by randomized controlled trials. Agglutination in cold agglutinin disease is IgM-mediated and independent from the complement system, but warm IgG autoantibodies can activate complement C. Since CBT treatment does not find the source of cold agglutinin production, it would be best if it is used as a “short course” to B-cell-directed therapy (6, 35). Complement overactivation and overconsumption should be reserved for devastating, life-threatening complement-related multiorgan failure with the need for short- or long-term complement blockade (6). Some authors believe that patients with severe AIHA who successfully respond to CBT may require long-term treatment (4). This was precisely why we looked at underground genetics, which may or may not support long-term CBT. The renewed AIHA relapse(s) can repeat complement activation with additional tissue and organ damage in an unfavorable underlying genetic constitution. We believe that complement regulators will terminate complement activation if warm and cold autoantibody stimulation is stopped. At the time of the AIHA onset, we did not have a C1 complement inhibitor at our disposal to increase the decreased C1q antigen levels (4). To the best of our knowledge, the aforementioned CBT inhibitors such as sutimlimab, Berinert, or pegcetacoplan have not been used in children. A well-thought-out decision by an experienced and skilled professional is necessary, and it should not be made on a hasty or random base (35).

We are aware of the effect that IVIg + B12 has on hemolysis AIHA control. In fact, it may be a late effect of rituximab/corticosteroid therapy, as the treatment success of combined warm and cold AIHA disease may take several months to respond. This is attributed to long-lived, non-proliferating plasma cells being resistant to chemo-/immunotherapy (24, 36, 37). For example, one patient with AIHA and Evans syndrome displayed a limited effect of one-dose IVIg after experiencing a prolonged prednisone therapeutic effect (3). Considering the long time lapse of the therapeutic effect of IVIg and prednisone therapy, the limited response of such therapy may be attributed to the lack of time for the disappearance of plasma cells from circulation (24, 36, 37). Nevertheless, the effect of anti-CD administration of IVIg on warm and cold AIHA should be completely acknowledged (3).

## Conclusion

We believe that the underlying disease was AIHA with cold and warm autoantibodies complicated with complement activation, which was probably triggered by a recent combination of EBV and CMV infection. The treatment of AIHA patients with warm and cold autoantibodies is challenging and should therefore be provided by following the recommended protocol. We administered corticosteroids, rituximab, vitamins B9 + B12, PLEX, and FFP. Final remission was achieved using PLEX and FFP. However, the fact of the disappearance of long-lived circulating plasma cells as additional late effects cannot be completely ignored. Complement activation should not be treated immediately with a complement blockade, except for case-sensitive complement overactivation and overconsumption, as most patients, after onset, should be amenable to self-complement regulation. Complement activation with a genetic background should be assessed in severe warm and cold hemolytic anemias caused by autoantibodies.

## Data Availability

The original contributions presented in the study are included in the article/Supplementary Material, and further inquiries can be directed to the corresponding author.
